# Knowledge development visualization and mapping path of the psychological capital research

**DOI:** 10.3389/fpsyg.2022.1064256

**Published:** 2022-11-17

**Authors:** Sun Meng, Xinwei Fu, Danxue Luo

**Affiliations:** School of Finance, Yunnan University of Finance and Economics, Kunming, China

**Keywords:** psychological capital, bibliometric visualization, burst detection, knowledge development, main path analysis

## Abstract

With the respect to the key factors, namely the psychological state of individuals and organizations, psychological capital (PsyCap) is widely used in various fields, such as management decisions and organizational behavior. To fully show the related studies and their knowledge development and mapping path, in this paper, we examine 2,786 papers about the PsyCap related research from 1970 to 2021. Based on the bibliometric analysis and main path demonstration (the tools are Cite-Space and Pajke, respectively), we derive some conclusions as follows: (1) the publication number about the PsyCap study is growing rapidly and it is a highly cross-cutting research topic. (2) The main authors come from Australia, the United States, and China, and also are the core researchers. (3) Refinement and measurement in the PsyCap study are constant and hot topics. (4) Stress, performance and well-being issues among students, health care workers and corporate employees are core research themes, and team organization, creativity, innovation, and COVID-19 are hot topics in this field. The bibliometric analysis are quantitatively analyzed to provide scholars with a more comprehensive insight into PsyCap research. The main path demonstration helps scholars to understand the main lines and key nodes of development in the field of psychological capital.

## Introduction

The core of Psychological Capital (PsyCap) is an individual’s integrated state of development in four psychological resources: Self-efficacy, hope, optimism, and resilience (Luthans et al., 2007; Luthans and Youssef-Morgan, 2017). Hope is defined as “a positive motivational state based on an interactively derived sense of successful, which includes agency and pathways” (Snyder et al., 1991). Efficacy is defined as “the individual’s conviction or confidence about his or her abilities to mobilize the motivation, cognitive resources or courses of action needed to successfully execute a specific task within a given context” (Stajkovic and Luthans, 1998; Luthans and Youssef-Morgan, 2017). Resilience is defined as “the capacity to rebound or bounce back from adversity, conflict, failure or even positive events, progress and increased responsibility” (Luthans, 2002; Luthans and Youssef-Morgan, 2017). optimism can be viewed as “an attributional style that explains positive events through personal, permanent, and pervasive causes and negative events through external, temporary, and situation-specific ones” (Peterson and Steen, 2002; Luthans and Youssef-Morgan, 2017). The growth in the number of PsyCap studies and the expansion of application scenarios has drawn the attention of scholars in other fields to PsyCap, especially in management and organizational behavior.

To promote and lead the healthy development of the field, many classic review articles have been developed, such as Dawkins et al. (2013), Anderson et al. (2014), Newman et al. (2014), Luthans and Youssef-Morgan (2017), and Nolzen (2018). They reviewed PsyCap in terms of background, concepts, theoretical mechanisms, measurement methods, and the current status of research, results, and applications. Also, they pointed out future research directions or gaps in existing research. But these articles tend to focus on a subfield to sort out, for example, Dawkins et al. (2013) concentrated on studies related to the concept and measurement of PsyCap. These researches hardly help beginners quickly understand the full picture of the development of the field (Zhou et al., 2022). Therefore, we employ bibliometric methods and main path analysis to systematically analyze the development trends of PsyCap research. The bibliometric approach provides a relatively complete network diagram of relationships (Li et al., 2020; Wang et al., 2020; Li and Xu, 2021), and measures the influence of authors, journals, institutions, and regions (Baumgartner and Pieters, 2003; Willett, 2007; Zhou et al., 2018), and detects classic literature and research hotspots (Su et al., 2021). For example, to show the knowledge mapping of Mobile learning and humanistic education research (Koon, 2022), present a visual analysis of research on digital transformation (Shi et al., 2022), display the research progress on innovation in the field of social capital (Gu et al., 2022), explore the development trend and frontier of sustainable logistics and supply chain (Wang et al., 2022), excavate the past, present, and future of the mindfulness field (Bunjak et al., 2022). It is also used to analyze the distribution and development of all literature within a given journal: To show the research progress of Mechanism and Machine Theory Journal from 1990–2020 (Flores, 2022), to summarize the research in the Journal of Fashion Marketing and Management (Kumar P. et al., 2022), to explore the emerging topic of European Management Journal (Bhukya et al., 2022).

There are many kinds of software for bibliometric analysis, such as SciMat (Cobo et al., 2012), VOSviewer (Van Eck and Waltman, 2010), CiteSpace (Chen, 2006), and so on. Each tool has its unique advantages. CiteSpace can perform citation bursts, which makes it more consistent with the research in this article. Therefore, this article mainly uses the tool CiteSpace. In addition, we further performed a master path analysis with the help the of Pajek tool, which helped to understand the main lines and important nodes of the development. For example, Yu and Pan (2021) used several different major paths to study the knowledge structure of TOPSIS and described its trends; Yu D. et al. (2022) explored the evolution of intuitionistic fuzzy set theory research themes using a master path analysis approach. Therefore, the results of both methods can be presented graphically, which can help the reader intuitively understand the salient features and changing trends in the PsyCap field. The contributions that this paper made are: (1) the *status quo*, the co-citation analysis, and the burst detection are quantitatively analyzed to provide scholars with a more comprehensive insight into PsyCap research; and (2) The inscription of the global standard main path, local forward main path, and local backward main path helps scholars to understand the main lines and key nodes of development in the field of psychological capital.

The rest of the paper is organized as follows: Section 2 describes the data sources and the specific bibliometric methods. Section 3 gives the results of four analysis types: basic statistical characteristics, collaborative network analysis, classical literature combing, and keyword analysis. Section 4 conducts the main path analysis. In Section 5, the conclusions are organized and research hotspots are discussed.

## Data sources and bibliometric methods

### Data source

We use the Web of Science (WOS), which is the most widely used tool by researchers (Falagas et al., 2008), to extract and gather reliable documents. To further ensure the quality of the documents, we select only two sub-databases in WOS, which are the Sciences Citation Index Expanded (SCI-Expanded) and Social Sciences Citation Index (SSCI). Then, we enter the search formula “TS = Psychological Capital” for the period 1900–2022 in the advanced search window. 2,786 documents were retrieved, corresponding to the time range 1970–2022.9.6. Finally, we export all the relevant information of the document from WoS in plain text format, including title, author, abstract, keywords, publications, and references.

### Analytical tool

We chose Cite Space (Chen, 2006), version 5.1.R8, the more commonly used software, to do the bibliometric analysis of PsyCap. Cite Space is simple to use and suitable for researchers to perform literature analysis quickly. It can precisely capture research hotspots, core authors, important institutions, and classic literature, as well as form clusters and detect bursts (Kleinberg, 2003), which helps scholars quickly grasp the development history and research hotspots in the field. The use of CiteSpace tools can be found in the classic literature, such as Fang et al. (2018); Pan et al. (2018), and Jiang et al. (2019).

In addition, we also apply Pajek to do the main path analysis about PsyCap, which was developed by Vladamir Batagelj from the University of Ljubljana. It is a complex network analysis tool (Batagelj and Mrvar, 1998) that helps to form the main path and sort out the most relevant literature (Olczyk, 2016). The specific information on the key nodes in the main path diagram can be obtained by HistCite Pro (Dan et al., 2021). For the use of Pajek in main path analysis, further references can be made to literature by Liu and Oakland (2016), Yu and Sheng (2020), Dong et al. (2022), and Yu Q. Y. et al. (2022).

## Data and methods

Based on the data and bibliometric methods described in the previous section, we further conduct an in-depth and comprehensive analysis of obtained document inWOS. The study was conducted in three dimensions: basic statistical characteristics, cooperation networks, and detection breakout points which focus on authors, institutions, countries or regions, cited literature, and keywords.

### Basic statistical characteristics related to psychological capital

#### Annual indicators of documents

**FIGURE 1:**
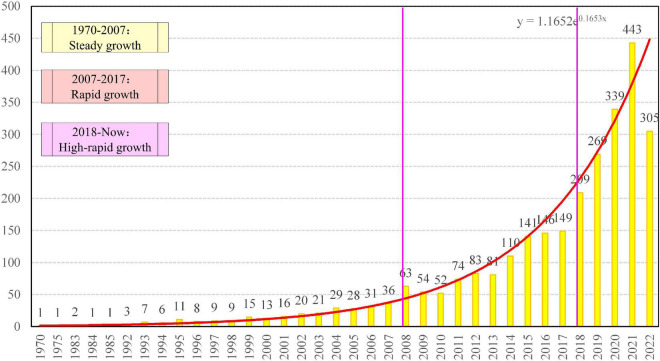
Growth of literature related to PsyCap, 1970–2022.

The number of publications per year broadly presents the research process in PsyCap. Therefore, we divide the research into three phases on its growth trend: Steady growth (1970–2007), rapid growth (2008–2017), and high-rapid growth (2018–2022.9), as shown in Figure 1.

In the first stage: Steady growth (1970–2007), there are a total of 268 documents. The first article was published in 1970, written by Glodber (1970), which focused on law psychology. In this stage, research is dedicated to the construction of PsyCap theory, and the theme gradually moved from macro-social issues to micro-intervention issues (Luthans et al., 2006). It is worth mentioning that the introduction of positive PsyCap measure methods (Luthans and Youssef, 2007) has contributed to the development of empirical studies.

In the second stage: Rapid growth (2008–2017), a total of 989 papers were published. During the decade, the introduction of core elements such as positive psychology (Avey et al., 2008), politics (Abbas et al., 2014), information technology (Burns et al., 2017), and measurement methods (Wernsing, 2014). Among the important research, objects are happiness, performance, satisfaction, education, and health.

The third stage: High-rapid growth (2018–2022.9). During this period, 1,565 papers were published. Team PsyCap (Tho and Duc, 2021), the mediating role of PsyCap (Kumar D. et al., 2022), and COVID-19 (Brunetto et al., 2022) have become important research themes, further contributing to the development of the field. The relatively small amount of literature for 2022 is caused by the fact that the specific date of the search data is September 9, 2022.

#### The most productive publications, categories, authors, affiliations, and countries/regions

**FIGURE 2:**
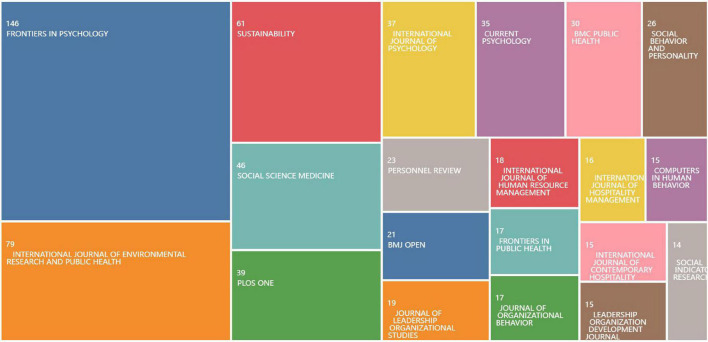
Visualization tree map of top-25 publications.

We obtain the main ten categories and publications from the WOS database, presented in Table 1 and Figure 2, respectively. And the literature covers 155 categories of research. From Table 1, we can get the top-10 research categories about PsyCap, which are “Psychology Multidisciplinary,” “Management,” “Public Environmental Occupational Health,” “Psychology Applied,” “Business,” “Environmental Sciences,” “Psychiatry,” “Sociology,” “Economics,” “Environmental Studies.” The total number and proportion of the literature in the top ten research categories were 2,318, and 83.23%, respectively, indicating that the research on PsyCap was relatively concentrated.

**TABLE 1:** The top 10 most-productive publications and authors.

Rank	Categories	Count	% of 2,786	Authors	Count	% of 2,786
1	Psychology multidisciplinary	434	15.58%	Luthans F	42	1.51%
2	Management	427	15.33%	Wang L	25	0.90%
3	Public environmental occupational health	383	13.75%	Avey JB	20	0.72%
4	Psychology applied	220	7.90%	Kawachi I	20	0.72%
5	Business	192	6.89%	Lindstrom M	20	0.72%
6	Environmental sciences	179	6.43%	Wang Y	15	0.54%
7	Psychiatry	155	5.57%	Li Y	14	0.50%
8	Sociology	113	4.06%	Liu L	13	0.47%
9	Economics	109	3.91%	Brunetto Y	11	0.40%
10	Environmental studies	106	3.81%	Kim M	11	0.40%

The top 25 publications shown in Figure 2, “Frontiers in Psychology,” “International Journal of Environmental Research and Public Health,” and “Sustainability” are the main publishers, with the number and percentage of publications (% of 2,786) being 146 (5.24%), 79 (2.84%), and 61 (2.19%). The journals ranked 4th to 10th are “Social Science Medicine,” “Plos One,” “International Journal of Psychology,” “Current Psychology,” “BMC Public Health,” “Social Behavior and Personality,” and “Personnel Review.” These journals aggregate a relatively large amount of research in the field of psychological capital.

According to the analysis of the productivity index, this paper further explores the concentration degree of PsyCap research at the author, institution, and country or region. Therefore, we present the top ten productive objects in Tables 1, 2.

**TABLE 2:** The top 10 most-productive affiliations and countries/regions.

Rank	Affiliations	Count	% of 2,786	Regions	Count	% of 2,786
1	University of London (England)	66	2.37%	United States	737	26.46%
2	University of Nebraska System (United States)	49	1.76%	China	594	21.33%
3	Harvard University (United States)	46	1.65%	Australia	261	9.37%
4	University of Nebraska Lincoln (United States)	44	1.58%	England	222	7.97%
5	University of California System (United States)	42	1.51%	Canada	124	4.45%
6	University of North Carolina (United States)	38	1.36%	South Korea	113	4.06%
7	University of Texas System (United States)	38	1.36%	Spain	99	3.56%
8	Monash University (Australia)	33	1.19%	Germany	94	3.38%
9	China Medical University (China)	32	1.15%	China-Taiwan	87	3.12%
10	Australian National University (China)	29	1.04%	Netherlands	75	2.69%

In Table 1, we can see that the top three authors are Luthans F (42), Wang L (25), Avey JB (20), Kawachi I (20), and Lindstrom M (20). Among them, Luthans F and Avey JB have more cooperation, and the research direction is the same, that is, positive PsyCap. Kawachi I focused on the relationship between social capital and mental health. Wang Y, Li Y, and Liu L are concerned with the moderating or mediating effects of PsyCap. Lindstrom M analyzes the role of PsyCap on the population-based. Brunetto Y is more interested in COVID-19 and innovation. Kim M introduces the element of PsyCap into the sports field.

Table 2 shows the top 10 most productive affiliations. They are the University of London, University of Nebraska System, Harvard University, University of Nebraska Lincoln, University of California System, University of North Carolina, University of Texas System, Monash University, China Medical University, and Australian National University. Among them, one is from England, six come from the USA, two are from Australia, and one is from China. We can be seen that the USA, China, England, and Australia are the most productive countries or regions.

### Cooperation networks among countries/regions, institutions, and authors

**FIGURE 3:**
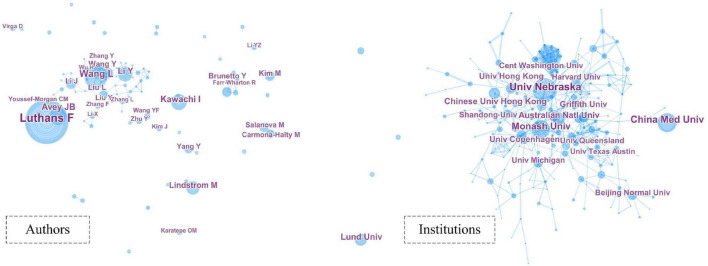
Collaboration network of authors and institutions.

Collaborative network analysis can help to understand the internal relationships of research in the PsyCap. Figure 3 shows the collaboration networks of authors and institutions. The size of the circle responds to the intensity of cooperation, and the larger circle indicates a higher frequency of cooperation and a stronger willingness to cooperate (Chen, 2006; Zhou et al., 2020). Table 3 shows the top 10 cooperative authors and institutions, authors are Luthans F, Wang L, Avey JB, Kawachi I, Li Y, Lindstrom M, Liu L, Wang Y, Brunetto Y, Li J. Among the top 10 most collaborative institutions, China Medical University, Chinese University Hong Kong, and University Hong Kong are from China; Monash University, Australian National University, and Griffith University are from Australia; University Nebraska and Central Washington University are from the United States; University Copenhagen is from Denmark, and Lund University is from Sweden. Analysis Tables 1, 3, we can find that more productive authors had more cooperative experiences.

**TABLE 3:** The top-10 most cooperative authors, institutions and regions/countries.

Rank	Authors	Count	Institutions	Count	Regions/Countries (centrality)	Count
1	Luthans F	39	University Nebraska	38	United States (0.44)	710
2	Wang L	20	China Medical University	30	China (0.12)	589
3	Avey JB	15	Monash University	29	Australia (0.15)	252
4	Kawachi I	14	Lund University	19	England (0.29)	218
5	Li Y	13	Australian National University	19	Canada (0.1)	118
6	Lindstrom M	12	Chinese University Hong Kong	18	South Korea (0.01)	109
7	Liu L	10	University Hong Kong	16	Spain (0.12)	92
8	Wang Y	10	University Copenhagen	16	Germany (0.09)	88
9	Brunetto Y	9	Griffith University	16	China-Taiwan (0.01)	82
10	Li J	9	Central Washington University	15	Japan (0.02)	68

Centrality is an indicator to measure the importance of nodes in the network.

Further, Table 3 also lists the top 10 most cooperative countries/regions, and summarizes the cooperate count and the centrality. Centrality is an indicator to measure the importance of nodes in the network. CiteSpace uses this indicator to find and measure the importance of a document (Freeman, 1979). Not surprisingly, the top three countries with the highest number of collaborations are the United States (710), China (589), and Australia (252), followed by England (218), Canada (118), South Korea (109), Spain (92), Germany (88), China-Taiwan (82) and Japan (68). As can be seen from Table 3, more than half of the countries have centrality values greater than 0.1, suggesting that they would be seen as crucial nodes with marked influence (Li C. et al., 2017; Li X. et al., 2017). The country with the highest centrality is the United States (centrality is 0.44), indicating that it has extensive exchanges with other countries or regions in the field of PsyCap. Further analysis reveals that countries or regions with more cooperation have a relatively greater centrality.

### Citation networks among authors and journal

According to WoS, a total of 7,693 authors have published literature related to PsyCap. Aiming to discover which scholars have made distinguished contributions in this field, we mapped the citation network of authors using Cite Space, as shown in Figure 6.

**FIGURE 4:**
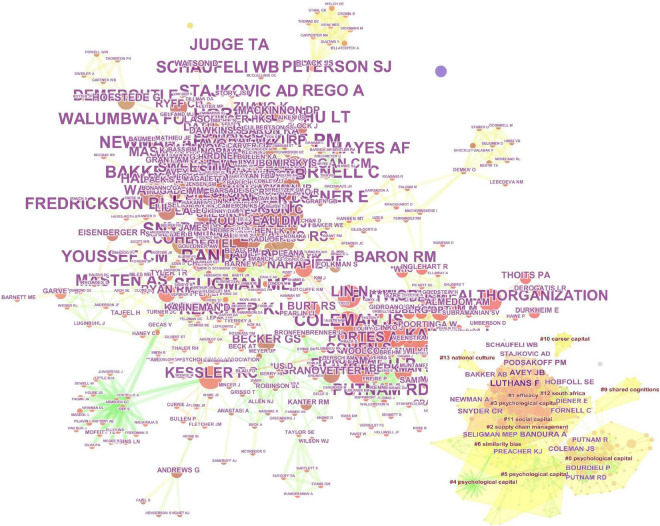
Citation network and cluster of authors.

**FIGURE 5:**
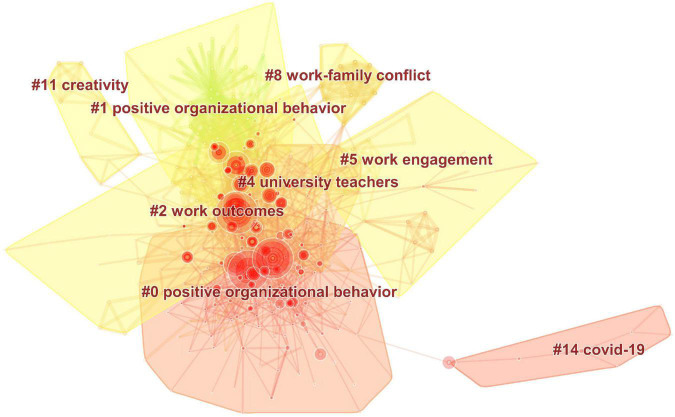
Co-citation network and the cluster of references.

**FIGURE 6:**
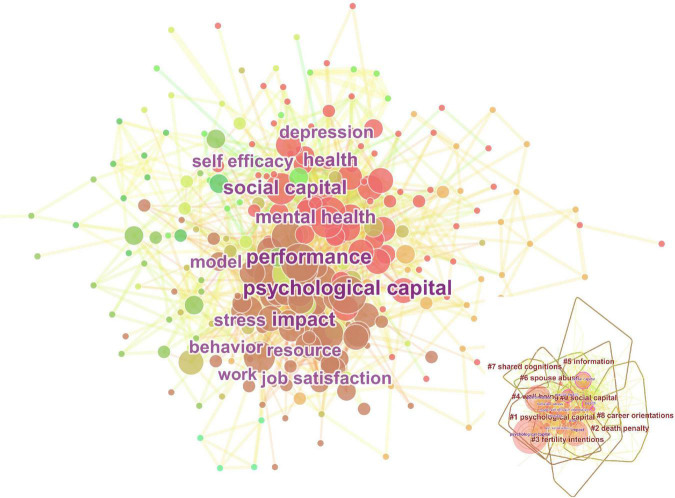
co-occurrence network of keywords.

Figure 4 contains two main elements: The large figure shows the network linkage graph between all authors; the small figure presents the clustering graph generated based on the authors’ citation networks. The twelve largest clusters that can be observed in the small figure are cluster “#0 PsyCap,” cluster “#1 efficacy,” cluster “#2 supply chain management,” cluster “#3 PsyCap,” cluster “#4 PsyCap,” cluster “#5 PsyCap,” “#6 similarity bias,” “#9 shared cognitions,” “#10 career capital,” “#11 social capital,” cluster “#12 south Africa” and cluster “#13 national culture.” Combined with Table 4, we can see that the top 10 most influential authors are Luthans F, Avey J B, Bandura A, Podsakoff PM, Hobfoll SE, Snyder CR, Coleman JS, Bakker AB, Bourdieu P, and Seligman MEP in the field of PsyCap. Among them, Luthans F, Avey JB, Podsakoff PM, and Bakker AB are the main authors in the cluster of “#3 PsyCap,” who are interested in positive PsyCap. Bandura A, Hobfoll S E, Snyder C R, and Seligman M E P worked on the cluster of “#2 supply chain management,” Coleman J S and Bourdieu P combined PsyCap with efficiency research. Additionally, Luthans F and Avey J B are also the most published and collaborated authors in this field.

**TABLE 4:** The top 10 most co-cited authors and journals.

Rank	Count	Authors	Count	Journal
1	971	Luthans F	934	Journal of Applied Psychology
2	619	Avey J B	912	Journal of Personality and Social Psychology
3	433	Bandura A	907	Journal of Organizational Behavior
4	400	Podsakoff PM	812	Personnel Psychology
5	347	Hobfoll S E	809	Journal of Management
6	311	Snyder C R	785	American Psychologist
7	240	Coleman J S	695	Psychological Bulletin
8	236	Bakker A B	672	Academy of Management Journal
9	234	Bourdieu P	593	Academy of Management Review
10	227	Seligman M E P	588	Social Science and Medicine

In Table 4, we can also find that the Journal of Applied Psychology is the most influential journal, with 934 citations in the field of PsyCap. From second to fifth places are the Journal of Personality and Social Psychology with 912 citations, the Journal of Organizational Behavior with 907 citations, Personnel Psychology with 812 citations, and the Journal of Management with 809 citations. The core journals that publish research papers on PsyCap are multidisciplinary or interdisciplinary scientific journals in sociology, medicine, management, and organizational behavior.

### Reference analysis and keyword analysis

#### Co-citation analysis of literature

The co-citation network can present the knowledge base of PsyCap research concretely. Therefore, we perform co-citation network analysis and clustering based on the Cite-Space tool for the 118,972 documents involved in this field, and the results are shown in Figure 5. Each node represents literature, and the size of the node corresponds to the number of literature citations. And the larger the node, the more citations the literature has received. The thickness of the connecting lines between nodes reflects the strength of association in the literature.

Table 5 lists the 10 most cited references sorted by the co-citation numbers. Specifically, the classical document that has the most co-citations is “Meta-analysis of the impact of positive PsyCap on employee attitudes, behaviors, and performance” by Avey et al. (2011), published in Human Resource Development Quarterly. This paper has 177 citations, which means that it plays an important role in the research of PsyCap. And this paper quantitatively analyzed the impact of PsyCap on employee attitudes, behaviors, and performance.

**TABLE 5:** The top-10 most cited references in PsyCap.

Rank	Title	Authors and years	Journal	Citation
1	Meta-analysis of the impact of positive psychological capital on employee attitudes, behaviors, and performance	Avey et al., 2011	Human Resource Development Quarterly	177
2	Psychological capital: A review and synthesis	Newman et al., 2014	Journal of Organizational Behavior	174
3	Psychological capital: An evidence-based positive approach	Luthans and Youssef-Morgan, 2017	Annual Review of Organizational Psychology and Organizational Behavior	170
4	Positive psychological capital: Measurement and relationship with performance and satisfaction	Luthans et al., 2007	Personnel Psychology	126
5	The development and resulting performance impact of positive psychological capital	Luthans et al., 2010	Human Resource Development Quarterly	113
6	Experimental analysis of a web-based training intervention to develop positive psychological capital	Luthans et al., 2008a	Academy Of Management Learning and Education	81
7	The additive value of positive psychological capital in predicting work attitudes and behaviors	Avey et al., 2010a	Journal of Management	79
8	Psychological capital: A positive resource for combating employee stress and turnover	Avey et al., 2009	Human Resource Management	77
9	Impact of positive psychological capital on employee well-being over time	Avey et al., 2010b	Journal of Occupational Health Psychology	76
10	The mediating role of psychological capital in the supportive organizational climate—employee performance relationship	Luthans et al., 2008b	Journal of Organizational Behavior	71

Rank at second place in the knowledge bases of PsyCap studies, the paper “PsyCap: A review and synthesis” is a classic literature review, authored by Newman et al. (2014), and published by the Journal of Organizational Behavior. This paper provided a summary of the literature on PsyCap, which provides an important reference for subsequent research. Thus, it has 172 citations.

The third-ranked literature is a literature review that comprehensively reviewed the measurement methods, theoretical mechanism, antecedents and consequences, analysis level, and research status of PsyCap. It has 170 citations, authored by Luthans and Youssef-Morgan (2017), and printed by the Annual Review of Organizational Psychology and Organizational Behavior.

The remaining seven articles are devoted to specific studies. Among them, Luthans et al. (2007) used resilience, optimism, effectiveness, and their composite indicators to predict job performance and satisfaction. Luthans et al. (2010) examined the impact of PsyCap interventions on human resource development and performance management. Luthans et al. (2008a) discovered that training interventions can develop positive PsyCap through a randomized trial. Avey et al. (2010a) identified the potential added value of PsyCap in predicting work attitudes and behaviors. Avey et al. (2009) studied the relationship between PsyCap and occupational stress, and found that PsyCap can counteract the negative effects of job stress. Avey et al. (2010b) examined the dynamic relationship between PsyCap and employee well-being. Luthans et al. (2008b) examined the relationship between PsyCap and employee outcomes. The results of the study showed that PsyCap was positively related to employee performance, satisfaction, and commitment.

Overall, the 10 most cited papers in PsyCap research are concerned about the effects of positive PsyCap on work behavior, work attitudes, and employee performance. Among them, there are four articles with Avey J B as the first author and five articles with Luthans F as the first author. These two authors have co-authored eight articles in the top 10 most cited references.

In addition, we further analyze the clustering information in Figure 5 can be found: In cluster “#0 positive organizational behavior,” Avey (2014), Newman et al. (2014), Youssef-Morgan and Luthans (2015), and Luthans and Youssef-Morgan (2017), are the crucial documents. And these documents are dedicated to sorting out and summarizing the theories, methods, and norms of PsyCap research from different directions. Nevertheless, cluster “#1 positive organizational behavior” is led by Luthans and Youssef (2007), Luthans et al. (2007), Luthans et al. (2008a), and so on, which focus on the impact of positive PsyCap on organizational behavior. In cluster “#2 work outcome,” literature such as Avey et al., 2010a,b and Avey et al. (2011), deeply explore the impact of positive PsyCap on performance. The cluster “#4 university teachers,” cluster “#5 work engagement,” cluster “#8 work-family conflict,” cluster “#11creativity,” and cluster “#14 COVID-19” are all core themes of PsyCap research, among which “COVID-19” is a new research hotspot.

#### Detection of burst points in co-cited literature

A systematic review and scrutiny of the relevant literature help to understand the progress of research, thus further complementing and refining the concepts and theories of PsyCap and standardizing research guidelines. These studies promote the healthy development of research in the field. Meanwhile, literature reviews usually analyze and summarize current research hotspots and future research directions, which to a certain extent point the way for research in the field. Therefore, there is no lack of literature review articles in PsyCap.

126 hot-cited references are obtained using burst point detection in the Cite-Space. We select these kinds of literature with the strongest citation burst ending in 2020–2022, displayed in Table 6, for a total of 45 articles. Analyzing them can further help us to understand the hot directions within the field of PsyCap in recent years.

**TABLE 6:** 45 References with strongest citation bursts (2012–2022).

Title	Years	Strength	Begin	End	2012–2022
Strength in adversity: the influence of psychological capital on job search	2012	4.41	2014	2020	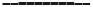
Work-family conflict and burnout among Chinese doctors: The mediating role of psychological capital	2012	5.92	2015	2020	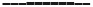
The impact of business school students’ psychological capital on academic performance	2012	7.94	2016	2020	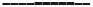
Sources of method bias in social science research and recommendations on how to control it	2012	12.13	2016	2020	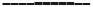
Authentic leadership promoting employees’ psychological capital and creativity	2012	14.21	2017	2020	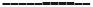
Psychological capital as a buffer to student stress	2012	4.91	2019	2020	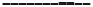
The impact of psychological capital on job embeddedness and job performance among nurses: A structural equation approach	2012	5.52	2019	2020	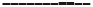
Building on the positives: a psychometric review and critical analysis of the construct of psychological capital	2013	6.76	2017	2022	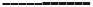
Meeting the leadership challenge of employee well-being through relationship psychological capital and health capital	2013	7.65	2017	2022	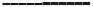
The relative trustworthiness of inferential tests of the indirect effect in statistical mediation analysis: Does method really matter?	2013	13.74	2018	2022	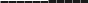
The impact of psychological capital on job burnout of Chinese nurses: The mediator role of organizational commitment	2013	5.12	2019	2020	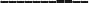
New nurses burnout and workplace wellbeing: The influence of authentic leadership and psychological capital	2014	6.04	2016	2020	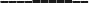
Psychological capital: A review and synthesis	2014	24.67	2017	2022	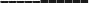
A critical review of the job demands-resources model: implications for improving work and health	2014	4.23	2018	2020	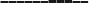
The left side of psychological capital: new evidence on the antecedents of psychology capital	2014	10.92	2018	2022	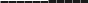
Psychological capital among university students: relationships with study engagement and intrinsic motivation	2014	13.27	2019	2022	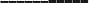
Innovation and creativity in organizations: a state-of-the-science review, prospective commentary, and guiding framework	2014	5.82	2019	2022	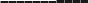
Building the leaders of tomorrow: the development of academic psychological capital	2014	6.99	2020	2022	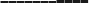
Getting to the “COR”: understanding the role of resources in conservation of resources theory	2014	11.11	2020	2022	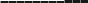
Do psychological capital and work engagement foster frontline employees’ satisfaction? A study in the hotel industry	2015	8.17	2017	2020	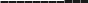
The impact of employees’ positive psychological capital on job satisfaction and organizational citizenship behaviors in the hotel	2015	7.87	2018	2022	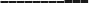
Why is hospitality employees’ psychological capital important? The effects of psychology capital on work engagement and employee morale	2015	11.57	2018	2022	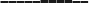
Psychological capital intervention (PCI): A replication and extension	2015	11.95	2019	2022	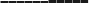
The relationship between psychological capital, job satisfaction, and safety perceptions in the maritime industry	2015	6.79	2019	2022	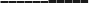
Impact of psychological capital on innovative performance and job stress	2015	10.78	2019	2022	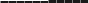
Linking positive emotions to work well-being and turnover intention among Hong Kong police officers: The role of psychological capital	2015	7.69	2020	2022	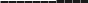
Effects of psychological capital on mental health and substance abuse	2015	9.59	2020	2022	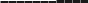
Toward better understanding of the learning goal orientation–creativity relationship: The role of positive psychological capital	2015	4.99	2020	2022	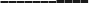
Psychological capital and well-being	2015	11.18	2020	2022	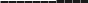
The mediating role of coping style in the relationship between psychological capital and burnout among Chinese nurses	2015	5.56	2020	2022	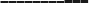
The mediating role of psychological capital on the association between occupational stress and job burnout among bank employees in China	2015	6.64	2020	2022	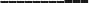
Why entrepreneurs often experience low, not high, levels of stress: The joint effects of selection and psychological capital	2016	7.63	2018	2022	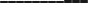
Psychological capital as a team phenomenon: mediating the relationship between learning climate and outcomes at the individual and team levels	2016	5.94	2019	2022	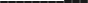
Job demands–resources theory: Taking stock and looking forward.	2017	10.47	2019	2022	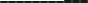
Psychological capital: An evidence-based positive approach	2017	47.50	2019	2022	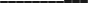
Psychological capital in the quick service restaurant industry: A study of unit-level performance	2017	5.94	2019	2022	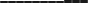
Test of a mediation model of psychological capital among hotel salespeople	2017	6.79	2019	2022	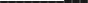
The cross-level mediating effect of psychological capital on the organizational innovation climate–employee innovative behavior relationship	2017	5.52	2020	2022	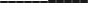
Psychological capital bolsters motivation, engagement, and achievement: Cross-sectional and longitudinal studies	2018	10.47	2019	2022	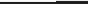
The concept of psychological capital: A comprehensive review	2018	9.24	2020	2022	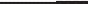
Psychological capital and performance among undergraduate students: The role of meaning-focused coping and satisfaction	2018	7.69	2020	2022	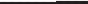
Testing a dynamic model of the impact of psychological capital on work engagement and job performance	2018	9.24	2020	2022	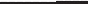
How psychological capital mediates between study-related positive emotions and academic performance	2019	4.61	2020	2022	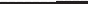
The psychological impact of quarantine and how to reduce it: Rapid review of the evidence	2020	8.08	2020	2022	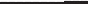
The psychological impact of the covid-19 epidemic on college students in China	2020	6.52	2020	2022	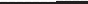

Dawkins et al. (2013) provided a comprehensive analysis and review of the theoretical conceptualization and psychometric properties of PsyCap and proposed six indicators to advance PsyCap research. Scholars have expanded the scope of empirical research on PsyCap by recognizing the emotional, cognitive, and motivational psychological states associated with creativity and innovation (Anderson et al., 2014). Thereafter, Newman et al. (2014) sorted out the factors affecting the development of PsyCap from the perspective of empirical analysis and the results of different levels of research. Halbesleben et al. (2014) expanded the theoretical study of PsyCap by combing the literature on resource conservation theory and suggesting the introduction of psychology and management. Underpinning these theoretical and empirical guides, the impact analysis, mediating effects, and practical applications of PsyCap have been further developed, with a comprehensive compendium and elaboration by Luthans and Youssef-Morgan (2017). With the focus on individual characteristics in the field of PsyCap, Nolzen (2018) called for researchers to further investigate the relationship between emotions and PsyCap, and suggests analyzing the effects of PsyCap in the context of strategic human resource management. In addition, Podsakoff et al. (2012) explored studies on the methodological bias, and Hayes and Scharkow (2013) recommend bias-corrected bootstrap confidence intervals as mediated analyses for the most trustworthy tests, which provide technical support for empirical studies of PsyCap.

The above meta-analysis of the literature shows that there has been much research on positive PsyCap and performance, behavior, and attitudes (e.g., satisfaction, commitment, happiness, and willingness to leave). Among them, the research based on individual objects: (1) the influential relationship or mediating role between employees’ PsyCap and job demands (Chen and Lim, 2012; Bakker and Demerouti, 2017), job satisfaction (Bergheim et al., 2015; Jung and Yoon, 2015; Karatepe and Karadas, 2015), well-being (Luthans et al., 2013; Youssef-Morgan and Luthans, 2015), performance/innovation performance (Abbas and Raja, 2015; Paek et al., 2015; Mathe et al., 2017; Alessandri et al., 2018), job stress (Laschinger and Fida, 2014; Li et al., 2015), and creativity (Rego et al., 2012; Huang and Luthans, 2015; Hsu and Chen, 2017); (2) the effect or mediating role of students’ PsyCap on academic performance (Luthans et al., 2012; Siu et al., 2014; Datu et al., 2018; Carmona et al., 2019), satisfaction (Ortega-Maldonado and Salanova, 2018), and stress (Riolli et al., 2012); (3) the moderating effect of PsyCap on health care workers on family conflict (Wang et al., 2012), performance (Sun et al., 2012), and job burnout (Peng et al., 2013; Ding et al., 2015).

Research based on teams and organizations, for example, Heled et al. (2016) explored the mediating effect of team PsyCap on learning climate, learning outcomes, job satisfaction, and team organizational behavior. Focus on the current special social environment, COVID is a research hotspot in the field of PsyCap. Brooks et al. (2020) found that after the outbreak of coronavirus disease in December 2019, the psychological impact brought by quarantine was extensive and substantial. Cao et al. (2020) studied the impact of COVID-19 on college students’ PsyCap.

Research on PsyCap metrics and nurturing interventions are also a research priority. Avey (2014) conducted two separate empirical studies of 1,264 engineers and technicians, and 529 Chinese scientists and technicians from an individual psychological perspective and found that individual differences, leadership ability, and job characteristics were strong predictors of PsyCap. Results from the Luthans et al. (2014) experimental study provide preliminary support that short-term training interventions can positively impact the academic PsyCap of business students. These two papers focused on short-term interventions for PsyCap, and Russo and Stoykova (2015) further explored the long-term effects of these approaches.

#### Keywords analysis

In bibliometrics, keyword co-occurrence is used to identify research trends. We employ Cite-Space to generate a keyword co-occurrence network for PsyCap, illustrated in Figure 6. Furthermore, we can find 9 clusters, which are formed based on the keyword co-occurrence network. The specific information on clustering is further presented in Table 7 and Figure 6.

**TABLE 7:** Co-occurrence of keywords in PsyCap.

cluster#0(77)	cluster #1(68)	cluster #2(29)	cluster #3(23)	cluster #4(20)	cluster #5(20)	cluster #6(13)	cluster #7(10)	cluster #8(4)
Social capital (356)	Psychological Capital (636)	Consequence (22)	Perception (65)	Stress (218)	Gender (38)	Women (24)	Network (49)	Labor (2)
Mental health (288)	Performance (510)	Psychological contract (11)	Experience (52)	Well-being (46)	Student (35)	United States (9)	Innovation (16)	Coping (2)
Health (279)	Impact (470)	Human resource management (9)	Children (35)	Commitment (31)	Human capital (27)	Preference (5)	Strength (2)	succe (2)
Depression (186)	Self-efficacy (214)	Identity (9)	Self (32)	Empowerment (17)	Education (24)	Gender difference (5)	Weak ty (2)	Flow (2)
Support (117)	Resource (201)	Intelligence (7)	Context (21)	Happiness (12)	Strategy (11)	Information technology (3)	Social structure (2)	

From the observation of Figure 6, we can see that cluster “#0 social capital” and cluster “#1 Psychological Capital” are the largest clusters, containing more keywords, and they are more closely connected. Cluster “#0 social capital” includes 77 keywords, cluster “#1 Psychological Capital” has 68 keywords, and cluster “#2 death penalty” contains 29 keywords. Cluster “#3 fertility intentions,” cluster “#4 well-being,” cluster “#5 information,” cluster “#6 spouse abuse,” cluster “#7 shared cognitions,” cluster “#8 career orientations” contain 23, 20, 20, 13, 10, 4 keywords, respectively. And the cluster “#8 career orientations” is the smallest cluster, which only has four keywords, including “labor,” “flow,” “succe” and “coping.” Table 7 lists the top five co-occurrence keywords with the highest frequency in each cluster.

Furthermore, we obtain 60 hot keywords using burst point detection in the Cite-Space (Ping et al., 2017). We highlighted the ten keywords with the greatest strength. The hot keywords generated during the steady growth (1970–2007) stage are “Self-rated health” (the strength is 11.21, begin at 2007, end and in 2016), “social capital” (10.58, 1999–2006), “Mortality” (10.3, 2000–2015) and “Women” (9.70, 2005–2015). The hot keywords generated during the rapid growth stage (2008–2017) are “Trust” (12.83, 2008–2016), “Positive organizational behavior” (11.42, 2010–2018), “Children” (10.64, 2012–2018) and “Organizational behavior” (9.96, 2010–2017). The others generated during the third stage (2018–2022) are “Nurse” (12.55, 2020–2022) and “Motivation” (9.97, 2019–2020). Noted that “Nurse” is a current hot topic of research in PsyCap. Among the related researches, the moderating effect of psychological capital on nurses in the workplace is a research priority. The literature related to the keyword “motivation” focuses on the relationship between psychological capital and the behavioral motivation of various workplace personnel, such as Datu et al. (2018), Xu et al. (2021), and Li et al. (2022).

## Additional discussions: The main path analysis

In this section, we use the main path analysis method to identify the main paths and the key nodes in the development of PsyCap. Specifically, we analyze the development of PsyCap knowledge from four perspectives: global key route, global standard route, local forward route, and local backward route.

**FIGURE 7:**
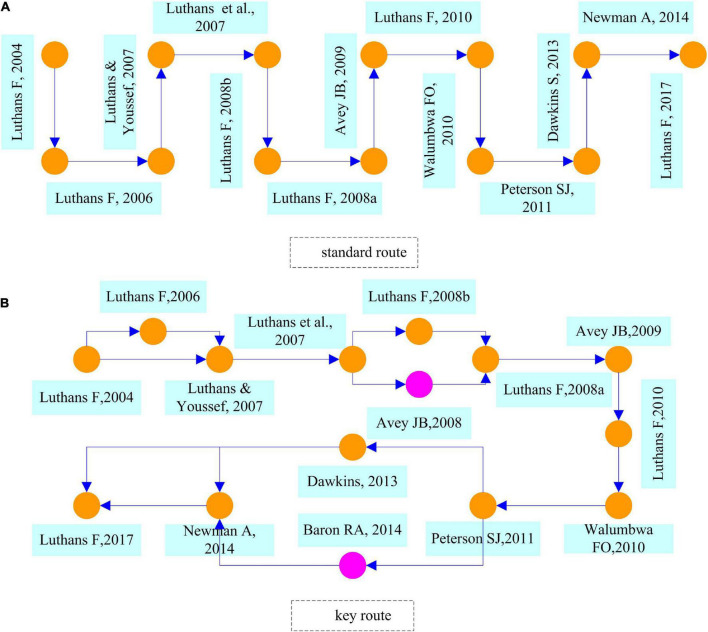
Global standard route **(A)** and key route **(B)** in the field of PsyCap.

As shown in Figure 7A, there is only one global standard route for PsyCap research, starting with Luthans and Youssef (2004), and ending with Luthans and Youssef-Morgan (2017). And one node represents one document and the arrows represent the direction of the study along the timeline. Luthans and Youssef (2004) argued that developing and managing employees’ positive PsyCap can improve their competitive advantage and suggested the following four channels: (1) developing self-efficacy/confidence, (2) developing hope, (3) developing optimism, and (4) developing resiliency. Luthans et al. (2007) analyzed how to develop a PsyCap approach from a micro-intervention perspective and explored the relationship between PsyCap on financial and investment reporting. Based on the concept of positive psychology (Seligman and Csikszentmihalyi, 2000) and related recommendations (Roberts, 2006) and guidelines (Kilduff, 2006) in organizational behavior, Luthans and Youssef (2007) combed through the literature on positive organizational behavior and laid the groundwork for research on how positive PsyCap affects organizational behavior.

The establishment of related theories has further led to the development of PsyCap research, from experimental (Luthans et al., 2007, 2008a,^2010^) to empirical (Avey et al., 2009), from impact analysis to mediated utility (Luthans et al., 2008b; Walumbwa et al., 2010), and from cross-sectional to longitudinal data (Peterson et al., 2011). In addition, scholars continue to sort out and summarize theories, methods, results, and applications in the field of PsyCap through literature reviews, among which the classic literature includes Dawkins et al. (2013), Newman et al. (2014), and Luthans and Youssef-Morgan (2017). This literature provides theoretical support and methodological bolster for subsequent studies.

Figure 7B shows the global key route in the field of PsyCap. There are 2 nodes in purple color, which means that two different documents are generated in the global standard route. Among them, Avey et al. (2008) have specifically studied the impact of positive PsyCap and positive emotions of employees on organizational change. Baron et al. (2016) study found that entrepreneurs have lower levels of stress that are attributable to their stronger PsyCap. And the correlation coefficient between corporate technical innovation and corporate leverage is negative in PsyCap.

**FIGURE 8:**
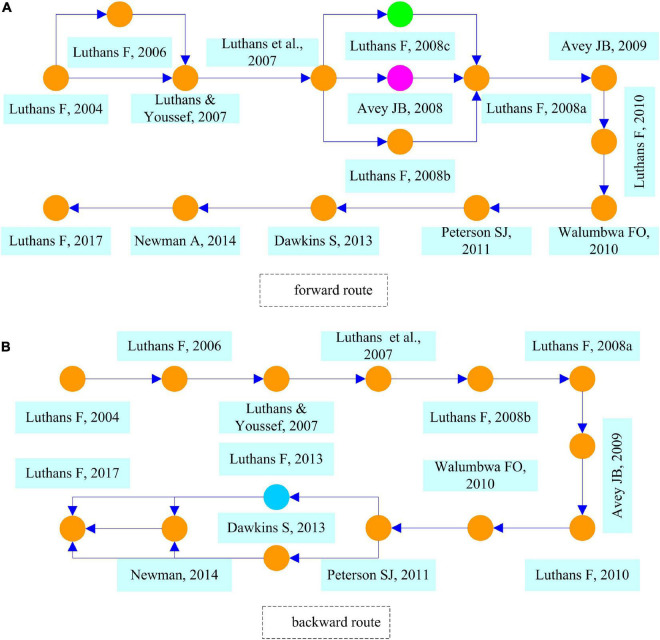
Local forward route **(A)** and backward route **(B)** in the field of PsyCap.

Figure 8A illustrates the local forward route in the PsyCap domain. Compared to Figure 7, we find a new node which is marked in green. This node represents the literature by Luthans et al. (2008c), which proposed an approach to human resource management based on PsyCap theory that applies to the Chinese environment.

Figure 8B displays the local backward route in the field of PsyCap. There are two main routes and 14 notes, and all paths converge to the node Luthans and Youssef-Morgan (2017) in the end. Meanwhile, a blue node is particularly conspicuous, and it corresponds to the literature that represents the extension of PsyCap to the field of well-being.

There are 17 documents in the four main paths mentioned above, of which five literature reviews lead and guide the development of the field. The remaining 12 essays are groundbreaking works, where PsyCap collides with each of the major themes. As PsyCap collided with other major themes, many seminal articles were produced, such as the 12 remaining articles in the main pathway.

## Conclusion

The statistics and analysis of the literature related to the field of PsyCap based on CiteSpace and Pajek provide a unique and meaningful snapshot of the subsequent research. Many conclusions can be drawn from the analysis: (1) The annual publication volume of the literature indicates that research in this field can be broadly divided into three phases: steady growth (1970–2007), rapid growth (2008–2017), and high rapid growth (2018–2022.9). According to the distribution of disciplinary classifications, it is clear that PsyCap has become an interdisciplinary study, mainly involving sociology, environmental studies, medicine, and business administration. Australia, the United States, and China dominate research on PsyCap, while these countries have the most prolific authors and institutions. (2) Grounded in the analysis of collaboration, a highly consistent relationship was found between authors who collaborated more and those authors who were more efficient. The same phenomenon was found in the countries or regions. And the overwhelming majority of the most influential journals are those in psychology and management. (3) Focusing on the clustering analysis of the cited literature, it is found that PsyCap research concentrates on the measurement and development of PsyCap and the influence or mediating role of PsyCap on organizational behavior and employee behavior. It is also interesting to note that the authors of the most cited literature are Luthans and Avey, who are likewise among the most prolific authors. (4) Based on the keyword clustering analysis and emergent point detection, it can be seen that in addition to the theme word “PsyCap,” performance, influence, health, social capital, and stress are the core keywords in this field; organizational behavior, nurses, and motivation have become hot topics in recent years. (5) The analysis of the four main paths reveals that the development of PsyCap research is as follows: theoretical construction to practical application, macro elaboration to micro empirical evidence, and static research to dynamic analysis. In addition, it is worth mentioning that the development of the psychological capital field is closely related to the influence and guidance of relevant policies, especially current research hotspots, such as innovation, well-being, and performance. Meanwhile, the improvement of psychological capital theories, methods, and norms, the expansion of the scope of empirical studies, and the improvement of the accuracy of the results have further improved the effectiveness of policy implementation.

We further summarized the current and future research hotspots in the field of PsyCap, as follows: (1) expand and refine the concept of PsyCap by incorporating other heart resources, such as emotional intelligence, courage, and forgiveness (Luthans and Youssef-Morgan, 2017); (2) The impact and mediating role of PsyCap on individuals, organizations or groups, from the direction of stress, motivation, and innovation, knowledge management; (3) Research on PsyCap development and the methods and effects of long- and short-term interventions; (4) cross-sectional research and use of PsyCap, for example, the impact of managers’ PsyCap on strategy and decision making (Nolzen, 2018).

## Data availability statement

The original contributions presented in this study are included in the article/supplementary material, further inquiries can be directed to the corresponding author.

## Author contributions

SM developed the theoretical framework. XF worked on data collection and processing. DL worked on literature review and manuscript writing. All authors contributed to the article and approved the submitted version.
